# The short-term effects of spiral stabilization on human posture correction

**DOI:** 10.1186/s13102-025-01218-y

**Published:** 2025-07-11

**Authors:** Xue Song, Zhe Li, Yang Song, M. Adeel Alam Shah, Xu-Hui Zhang, Chan Li, Campbell Gilmore, Wen-Bin Jiang, Hong-Jin Sui

**Affiliations:** 1 Department of Anatomy, Dalian Medical University, Dalian, 116044 China; 2 Department of Anatomy, Guang Dong Medical University, Dong Guan, 523808 China; 3 School of Management, Liaoning University of International Business and Economics, Dalian, 116052 China; 4 Department of Human anatomy, School of Health and Life Sciences, University of Health and Rehabilitation Sciences, Qingdao, 266071 Shandong China; 5 Medical school, St. George’s University of London, SW17 0RE London, United Kingdom

**Keywords:** Body posture, Spiral stabilization, Functional exercise, Fascial tissues

## Abstract

**Background:**

Changes in human posture directly impact the structures of various body parts, often leading to musculoskeletal disorders. While identifying suitable treatments for pain associated with long-term postural abnormalities is important, preventing such conditions is demonstrably a superior solution. Spiral stabilization, known for its practical application, has proven effective in treating low back pain. However, its efficacy in correcting human posture warrants further validation.

**Methods:**

A total of 71 participants with abnormal body posture, with a mean age of 33.68 ± 6.78 years, were included in this research. The participants underwent spiral stabilization practice for one hour daily for four days. The posture-related angles and deviations from the anterior and lateral views were calculated by the Exbody musculoskeletal analysis system.

**Results:**

There are statistically significant improvements in most posture-related angles and deviations after the intervention of the spiral stabilization technique compared to before the intervention (*P* < 0.05).

**Conclusion:**

The findings of this study suggest that the spiral stabilization technique is a potential intervention for improving human posture. It may become an effective fitness exercise that is widely adopted in daily life to prevent postural abnormalities.

**Trial registration:**

Registration date is July 10, 2021. The registration number is ChiCTR2100048568.

## Introduction

Body posture is generally defined as the alignment of body segments in an upright position [1]. Maintaining optimal body posture is essential for the health of both the musculoskeletal and motor systems. Studies indicate that proper posture control not only helps reduce the incidence of musculoskeletal diseases but also improves overall quality of life and functional independence [2].

‌ Abnormal posture refers to the deviation of the body from its normal physiological position in either a static or dynamic state, such as neck deviation from the midline, uneven shoulders, anterior pelvic tilt, and knee varus or valgus [1]. Such postural changes can negatively impact activities of daily living and health-related quality of life, potentially leading to a range of pain symptoms in severe cases [3]. For instance, in aging populations, postural changes can result in a decline in physical function [4]. Among athletes, particularly elite athletes, maintaining correct posture is deeply connected to a reduced risk of lumbar-related diseases [5]. In occupations involving prolonged sitting, such as office work, poor posture and extended sitting times can increase muscle stiffness in the back [6]. Furthermore, poor postures, including reading while lying down [7], incorrect sitting postures when using electronic equipment, increased pelvic obliquity [8], and reduced thoracic kyphosis length [9], can contribute to the occurrence of chronic low back pain (LBP) [10].

Currently, various treatment methods exist to improve body posture and alleviate pain associated with long-term postural abnormalities. For example, leg length discrepancy equalization treatment has been shown to significantly enhance postural parameters and reduce pain, with numerical pain rating scale scores decreasing from 7.8 to 0 in patients with nonspecific low back pain [11]. Aerobic exercise, resistance training, and McKenzie therapy are also exercise strategies used to manage chronic LBP [12, 13]. Some of these treatments necessitate the expertise of a physical therapist, involve site-specific limitations, and can be costly in terms of time and resources. While finding appropriate treatments for pain associated with long-term postural abnormalities is important, discovering ways to prevent these issues is an even more beneficial solution. If such a prevention method can be performed independently and cost-effectively, it would be highly advantageous.

**Fig. 1:**
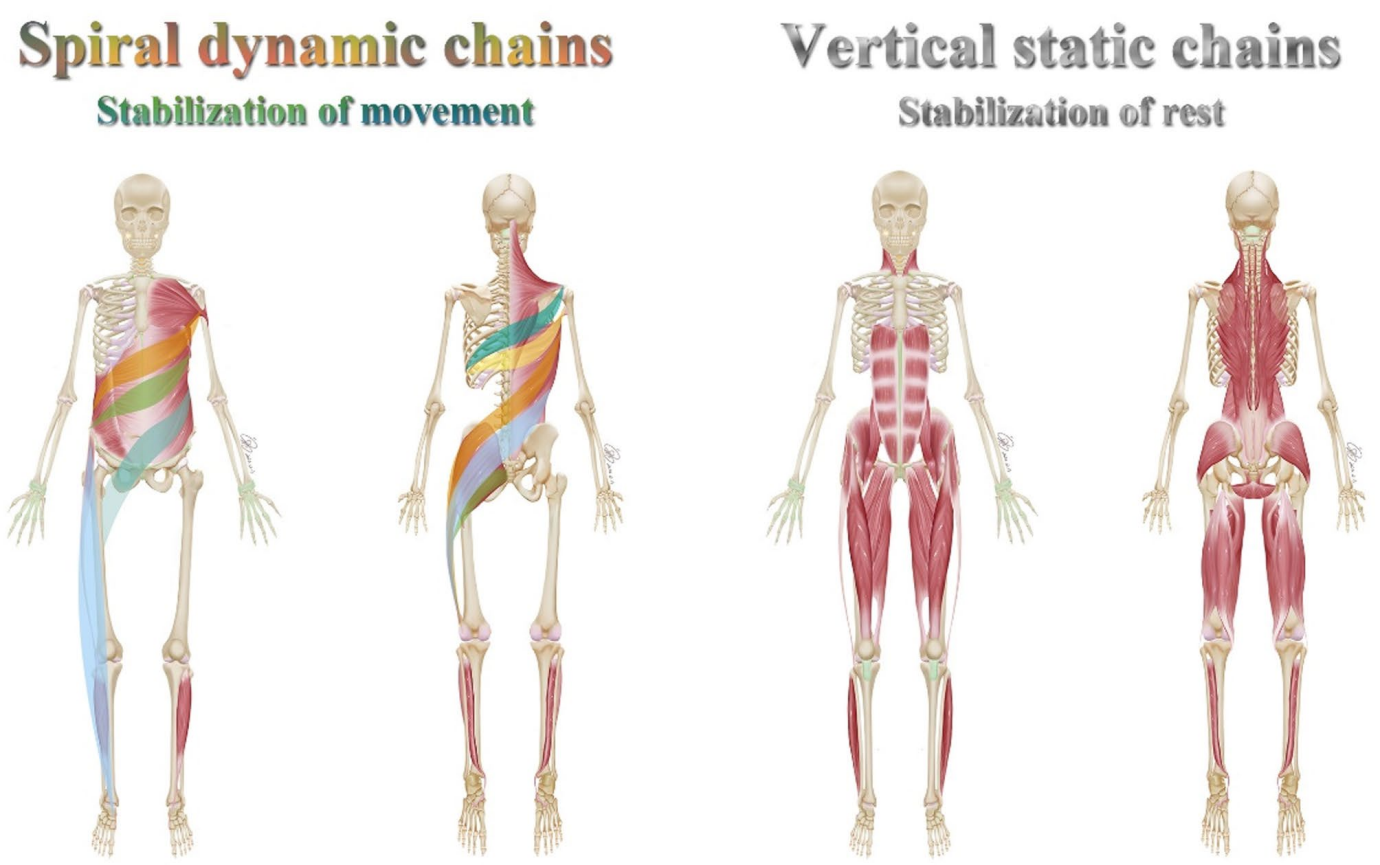
The spiral dynamic chain and the vertical static chain. The spiral dynamic chain is mainly composed of the latissimus dorsi, trapezius, serratus anterior, and pectoralis major muscles. The vertical static chain consists of erector spinae muscle, quadratus lumborum muscle, rectus abdominis muscle, and iliopsoas muscle

Recently, fascial tissue treatments have gained popularity in addressing musculoskeletal issues [14, 15]. Fascia is considered a critical component of a tensegrity-like, body-wide network, functioning as a linking structure that prevents muscles from acting as independent units [16]. Fascia can transmit tension [17, 18] and possesses proprioceptive [19] and nociceptive functions crucial for adapting to strain and maintaining normal musculoskeletal dynamics [20]. The Czech doctor Richard Smisek developed the Spiral Stabilization Muscle Chain (SS) technique, which incorporates both a spiral dynamic chain and a static vertical chain, connecting distant parts of the body through muscles and fascial tissues (Fig. 1) [21]. This technique has been widely used for musculoskeletal intervention and has been popular for 40 years in countries such as the Czech Republic, Germany, Korea, and China, owing to its convenience, simplicity, and effectiveness. A recent clinical study demonstrated the effectiveness of the SS technique in treating patients with LBP [22]. However, further evidence is needed to determine the impact of the spiral stabilization system on body posture correction.

The Exbody musculoskeletal analysis system automatically records participants’ body postures from various photographic views, enabling accurate identification of posture-related parameters [23]. An overall deviation value exceeding 10 in the Exbody musculoskeletal analysis system indicates the onset of body imbalance. Therefore, the objective of this article is to investigate the role of the SS technique in correcting body posture by evaluating changes in posture-related parameters using Exbody software. By integrating and coordinating information from the entire body, SS technology will aid in developing a series of targeted exercises to address or prevent musculoskeletal disorders associated with poor posture.

## Materials and methods

### Ethical approval details

This study was approved by the ethics committee of Dalian medical university.

### Study design

This study was a pre- and post-intervention clinical trial registered with the Chinese Clinical Trial Registry (registration number: ChiCTR2100048568). A single-blinded study design was employed, with no communication between the observer and therapist regarding the research details. Participants were blinded to the study hypotheses. The study assessed the body posture deviation of each participant with potential posture abnormalities before and after the intervention. All participants provided informed consent and the study adhered to the principles outlined in the Declaration of Helsinki.

### Sample size

This study was unable to calculate the sample size because there is no literature, to our knowledge, on the treatment of body posture with the SS technique, and no preliminary data. This was an exploratory experiment, and the sample size consisted of 71 participants who were ultimately recruited and met the criteria.

### Study participants

71 volunteers with potential posture abnormalities were selected to participate in our training of the SS technique. Participants who met the following criteria were included in the study: (A) value of overall deviation > 10 before intervention technique, as measured using the Exbody system, suggesting the body in an unbalanced state; (B) aged 18 years or older, able to ambulate independently, no leg-length discrepancy, no spinal pathology or deformity, and no history of hip, pelvis, or lower-limb disorders. Individuals with a history of lower back, hip, or knee pain lasting three or more consecutive months were excluded. We also collected demographic characteristics, including age and gender. Informed consent was obtained from all patients for the publication of the case report details.

### Intervention techniques

**Fig. 2:**
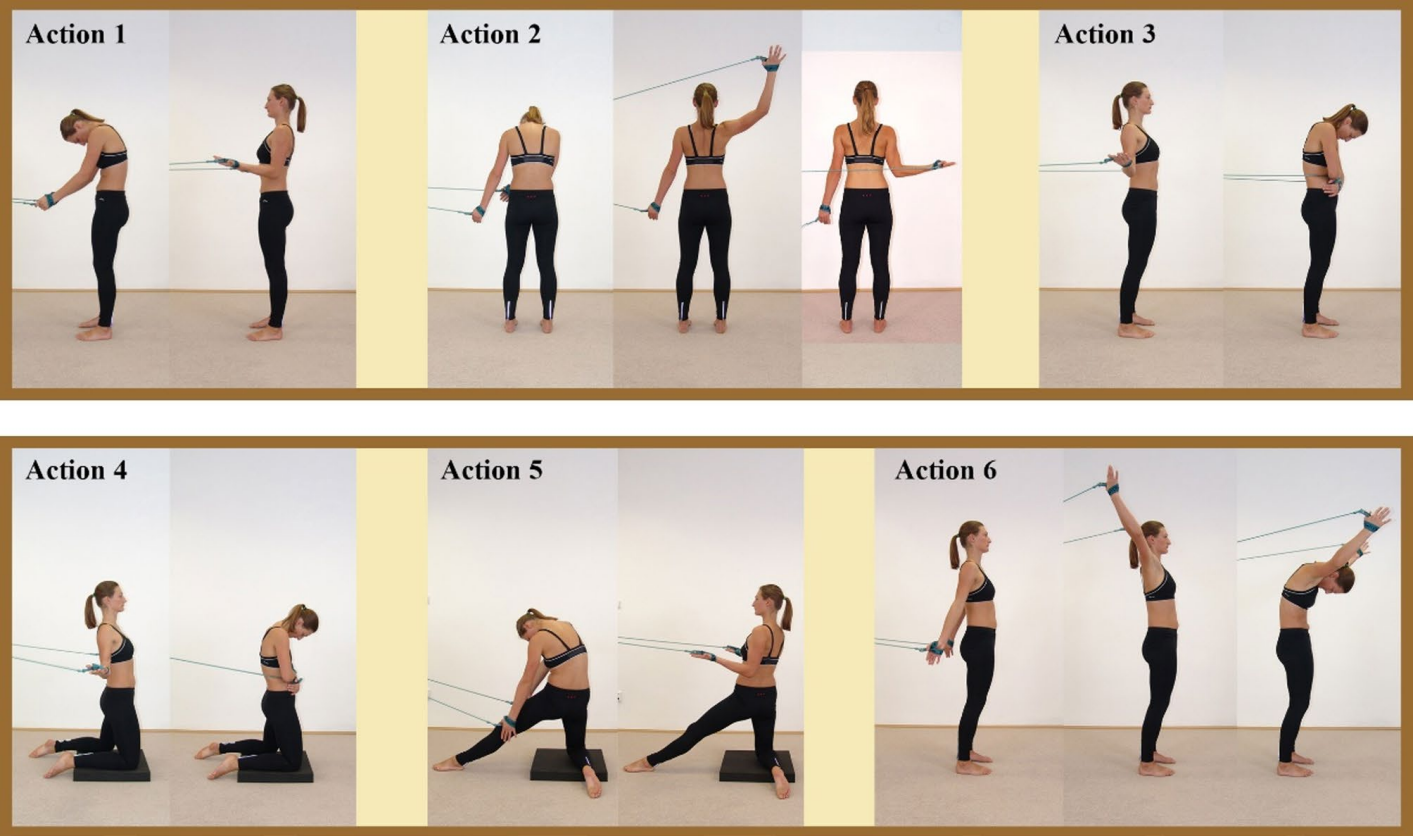
The spiral stabilization (SS) technique. In Action 1, the latissimus dorsi muscle is activated. In Action 2, the trapezius muscle is activated. In Action 3, the pectoralis major muscle is activated. In Action 4, the pectoralis major and gluteus maximus muscles are activated. In Action 5, the latissimus dorsi and gluteus maximus muscles are activated. In Action 6, the serratus anterior muscle is activated. (Images from: https://www.spiralstabilization.com/en/)

The intervention training for the SS technique is illustrated in Fig. 2. All participants began in a neutral standing position. An exercise cord, specifically designed for spiral stabilization exercises, was attached to the participant’s hand to assist in completing all SS technique actions while also regulating breathing (Fig. 2).

The actions were performed as follows:

Action 1: Stand on both legs and pull the exercise cord backward with both arms.

Action 2: Stand with both legs and pull the exercise rope sideways with one arm.

Action 3: Stand on both legs, spread the arms backward, and then bilaterally adduct the shoulder joints.

Action 4: Kneel on both knees with the pelvis slightly forward, and repeat action 3.

Action 5: Kneel on one knee, extend one leg forward, and reach backward in a spinal protrusion position. Use both arms to pull the exercise cord backward.

Action 6: Stand naturally with lower limbs and wrap arms forward. and pull the exercise cord with both arms from behind, up, and then forward.

These actions activated the latissimus dorsi, trapezius, pectoralis major, gluteus maximus, and serratus anterior muscles. All actions were performed under the guidance of two professional experimenters, who assisted participants in completing them independently. Experimenters instructed participants to focus on the sinking and retraction of the shoulder blades, the upward movement of the head, and the backward and downward sensation of the pelvis, among other cues.

Training started with action 1 and progressed gradually. Each action was performed 10–15 times per set, with a 2-minute interval between sets, three times daily. Each participant underwent one hour of SS practice daily for four consecutive days.

### Photographs and measurements

Standard protocol was followed for photographs and measurements of the anterior and lateral views, with participants standing upright using the Exbody 650 version 1.0 (Huimao Technology, Inc., Beijing, China). For the lateral view, participants were instructed to stand naturally with feet together on flat ground, avoiding any forced position, while gently placing their right hand on their navel with the forearm positioned approximately 90° to the vertical.

**Fig. 3:**
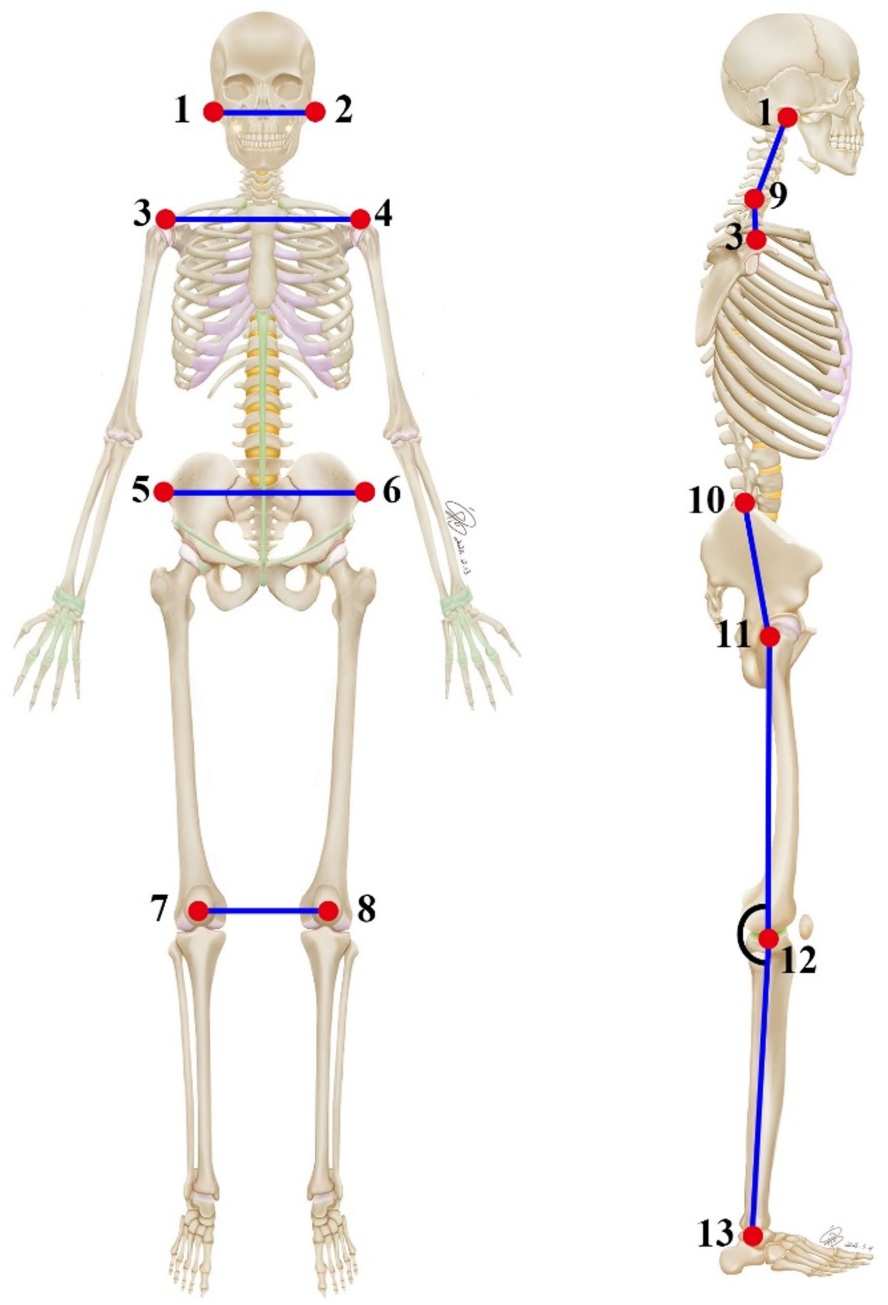
Anatomical points of the anterior and right lateral views. (**1, 2**) Tragus; (**3, 4**) acromion; (**5, 6**) anterior superior iliac spine; (**7, 8**) patellar; (**9**) the midpoint of the neck; (**10**) the highest point of the ilium; (**11**) greater trochanter; (**12**) the midpoint of the knee joint; (**13**) lateral malleolus

As mentioned in the previous literature [24], thirteen anatomical reference points were selected from the anterior and right lateral views (Fig. 3). These points were identified and marked by experienced anatomy instructors to minimize machine error and ensure the accuracy of the marked points. Subsequently, the specialized Exbody software was used to capture digital photographs and calculate posture-related angles from different views, as shown in Fig. 3; Table 1. Finally, the results for front deviation and lateral deviation were automatically generated using the specialized Exbody system. Overall deviation is the sum of front deviation and lateral deviation. Table 1 describes the measurement method of the assessed angles (degrees) and deviations, along with their interpretation.

**Table 1:** Description of the measurement method of the angles (Degrees) and deviations assessed and their interpretation

Views	Acronym	Method (Based on Fig. 3)	Definition of Angles or Deviations	Interpretation of Angles or Deviations Values
Anterior view	HAH	Angle between points 1–2 and horizontal	Horizontal alignment of head	Positive values indicate tilting to the right, while negative values indicate tilting to the left.
	HAA	Angle between points 3–4 and horizontal	Horizontal alignment of acromions	
	HAASIS	Angle between points 5–6 and horizontal	Horizontal alignment of anterior superior iliac spine	
	HAP	Angle between points 7–8 and horizontal	Horizontal alignment of patella	
Right lateral view	VAH	Angle between points 1–9 and vertical	Vertical alignment of head	Positive values indicate anterior position, while negative indicate posterior position.
	VAS	Angle between points 3–9 and vertical	Vertical alignment of shoulder	
	PA	Angle between points 10–11 and vertical	Pelvic angle	
	KA	Angle between points 11-12-13	Knee angle	Values > 180°: indicates knee hyperextension; Values < 180°: indicates knee flexion.
Anterior view	FD	Automatically generated by the Exbody system	Front deviation	A higher value indicates worse body posture.
Right lateral view	LD		Lateral deviation	
both views	OD		Overall deviation	

Anatomical points. (1, 2) Tragus; (3, 4) acromion; (5, 6) anterior superior iliac spine; (7, 8) patellar; (9) the midpoint of the neck; (10) the highest point of the ilium; (11) greater trochanter; (12) the midpoint of the knee joint; (13) lateral malleolus

### Statistical analysis

Data concerning the assessed angles and deviations, both before and after the intervention, were processed and analyzed using IBM SPSS Statistics version 25. The Kolmogorov-Smirnov test (number of volunteers > 50) was conducted to assess normal distribution. Measurement data conforming to a normal distribution were expressed as mean ± standard deviation and compared using the paired t-test. Data not conforming to a normal distribution were expressed as median (interquartile range) and compared using the non-parametric Wilcoxon signed-ranks test. A *P*-value of less than 0.05 was considered statistically significant.

## Results

A total of 71 participants were included in the study, ranging in age from 18 to 51 years, with a mean age of 33.68 ± 6.78 years. The sample comprised 5 men and 66 women. These participants exhibited an overall deviation value greater than 10, as measured by the Exbody system prior to the intervention, indicating a state of body imbalance. The data for posture-related parameters, both before and after the SS technique intervention, were found not to follow a normal distribution. Therefore, the data are presented as medians, and the non-parametric Wilcoxon signed-ranks test was utilized for analysis.

Table 2 presents the posture-related angles used for statistical analysis with the Exbody software. From the anterior view, the angle values of HAH, HAA, HAASIS, and HAP showed a significantly downward trend post-treatment compared to pre-treatment (*p* < 0.0001), suggesting improved horizontal balance of the head, acromions, anterior superior iliac spine, and patella (Table 2). From a right lateral view, the values of VAH and PA distinctly decreased after treatment compared to before treatment, indicating significant improvement in forward head posture and pelvic tilt symptoms (Table 2). However, no statistically significant differences were observed in the values of VAS and KA before and after the intervention (Table 2).

**Table 2:** Comparison of the posture-related angles between pre- and post-intervention results of the spiral stabilization technique

Angles	pre-intervention (°)	post-intervention (°)	*P*-value	Effect Size
HAH	0 (0, 3)	0 (0, 0)	< 0.05	**+**
HAA	1 (1, 2)	1 (0, 2)	< 0.01	**++**
HAASIS	2 (1, 3)	1 (0, 1)	< 0.0001	**++++**
HAP	2 (1, 2)	0 (0, 1)	< 0.0001	**++++**
VAH	7 (4, 11)	5 (3, 9)	< 0.01	**++**
VAS	1 (1, 2)	1 (1, 2)	>0.05	**−**
PA	6 (1, 10)	3 (1, 7)	< 0.05	**+**
KA	179 (176, 182)	179 (177, 182)	>0.05	**−**

Data are presented as the median (interquartile range). *P* values were calculated using the Wilcoxon signed-rank test. **−**: no effect; **+**: poor effect; **++**: moderate effect; **+++**: good effect; **++++**: very good effect

**Fig. 4:**
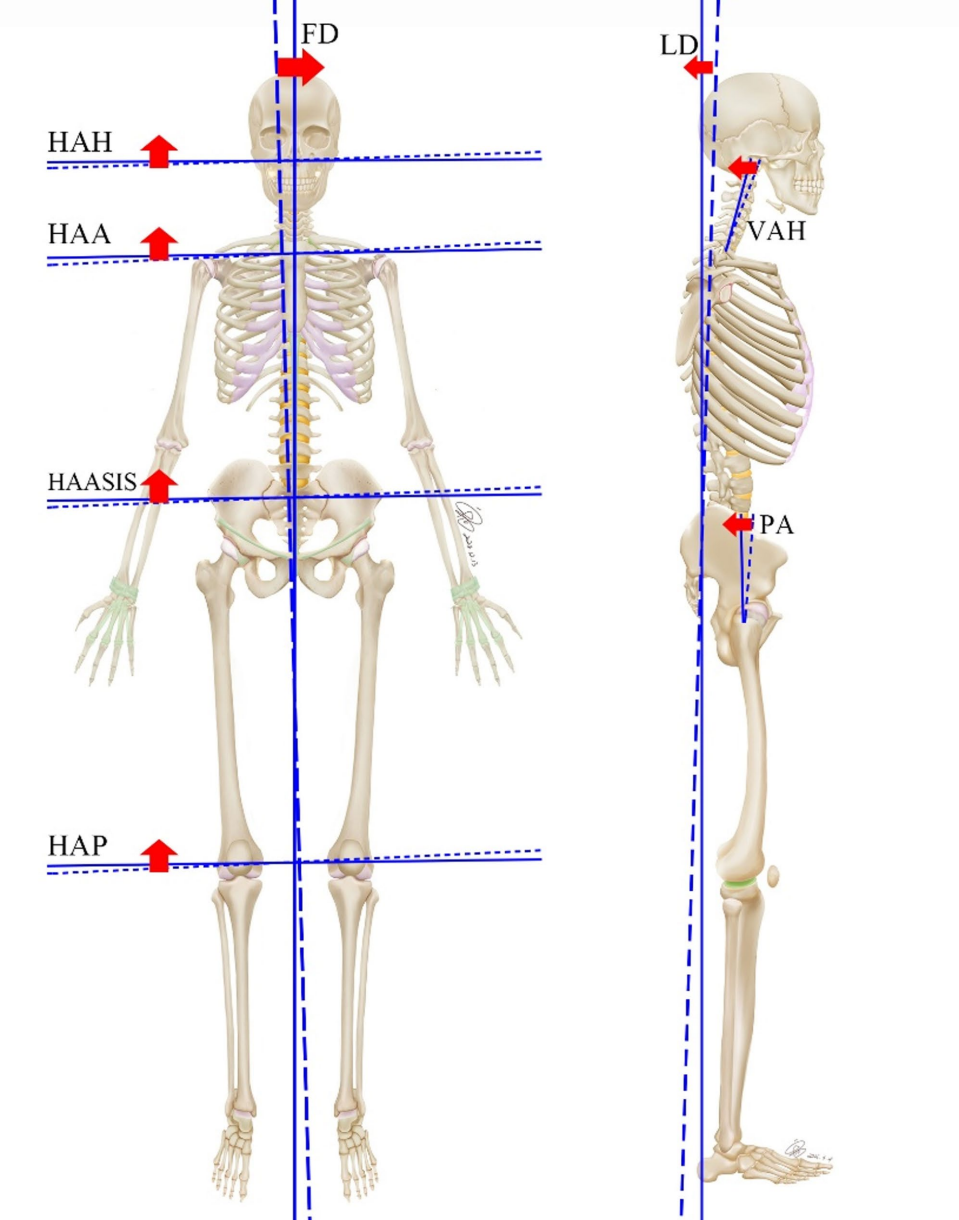
The visual presentation highlights the key indicators of spiral stabilization technique for body posture correction. The dotted line represents the parameter state before the intervention. The solid line represents the parameter state after the intervention. The red arrow represents the positive effect of the indicators. HAH: Horizontal alignment of head; HAA: Horizontal alignment of acromions; HAASIS: Horizontal alignment of anterior superior iliac spine; HAP: Horizontal alignment of patella; VAH: Vertical alignment of head; PA: Pelvic angle; FD: Front deviation; LD: Lateral deviation

Table 3 displays the results of the lateral, frontal, and overall deviations automatically generated by the Exbody system. Statistical analysis of the front, lateral, and overall deviations before and after the SS technique intervention revealed a statistically significant difference (*p* < 0.0001), indicating that the SS technique significantly improved body posture (Table 3). Figure 4 further highlights the key indicators of spiral stability used for human posture correction through visual presentation.

**Table 3:** Comparison of frontal, lateral, and overall deviations between pre- and post-intervention results of the spiral stabilization technique

Parameters	pre-intervention	post-intervention	*P*-value	Effect Size
Front deviation	8 (5, 12)	4 (2, 7)	< 0.0001	**++++**
Lateral deviation	18 (14, 23)	14 (11, 17)	< 0.0001	**++++**
Overall deviation	27 (22, 33)	18 (15, 23)	< 0.0001	**++++**

Data are presented as the median (interquartile range). *P* values were calculated using the Wilcoxon signed-rank test. **++++**: very good effect

## Discussion

Maintaining an upright posture requires both muscle strength and proper alignment of bones and joints [25]. Any physiological or pathological issues in the trunk or legs can disrupt this alignment and overall postural balance, leading to compensatory changes in other segments. This, in turn, can destabilize the body and result in a loss of balance. Therefore, restoring the counterbalance between the dorsal and ventral muscle chains is crucial for improving body posture [26]. The present results demonstrate that the spiral stabilization (SS) technique can significantly improve the horizontal and vertical imbalance of the head, shoulders, pelvis, and knee joints, as well as significantly reduce lateral, front, and overall body deviations.

Pavol et al. reported that the SS technique had a comparable effect on reducing disability and improving the management of daily activities and physical functions [22]. The present study further reveals that the SS technique may effectively improve joint alignment and reduce deviations. According to the theoretical system of the SS technique [27], the latissimus dorsi, trapezius, pectoralis major, gluteus maximus, and serratus anterior muscles were activated in the present study, while also providing a slight stretch to the paraspinal and pelvic muscles. Under the influence of internal stretching, the instability of the spine and joints may be alleviated. Due to the enhanced constraint of the spiral muscle chain, the human musculoskeletal system is able to maintain good postural tension, thereby facilitating better postural recovery. These factors may explain the improvement in individual postural imbalance observed in the results of study, encompassing the head and neck, shoulders, hips, knees, and spine. In short, the SS technique improves overall posture control and stability by optimizing muscle activation patterns, especially for individuals who require exercise.

However, two non-significant indicators were observed in this study, which is an unsatisfactory outcome. It is hypothesized that the intervention time of the SS technique may have been too short, and the muscles directly related to the SS technique are primarily located in the chest, waist, and pelvis. Longer SS technique interventions may be needed to better correct postural abnormalities in the neck and lower limbs.

The skeletal muscles of the entire body are indirectly connected through fascial tissues, forming a network with a specific pattern, later termed the ‘myofascial meridian’ by Myers [28, 29]. These myofascial chains are a viscoelastic substance that enhances centrifugal control at the extremities of joint movement [30]. Moreover, myofascial chains possess the ability to generate and transmit passive tension [17, 18], which is essential for spontaneous adaptation and adjustment to strain or stretch stress, thereby maintaining normal musculoskeletal dynamics [20]. The SS technique is based on the realization of the synergic work from the musculoskeletal and fascial systems [21, 27]. As a widely applicable, low-cost intervention, the SS technique helps to improve muscle strength, coordination, and stability through specific movement patterns.

Improvements in body posture can, in turn, directly influence muscle activation patterns, reducing compensatory activation in non-target muscle groups and the generation of trigger points [31]. The muscles activated by the SS technique contain muscle spindles, which are responsible for sensing the length and speed of muscle stretch and play a crucial role in maintaining muscle tone and transmitting proprioceptive information [32, 33]. Fascial tension altered by activating or modifying the function of these muscles affects the mechanical coupling efficiency of muscle spindles and regulates proprioceptive feedback [34]. Ultimately, this will improve body posture and thereby reduce perceived discomfort.

Currently, various treatment methods aim to improve human posture, such as the Digital Posture Assessment and Correction System, which evaluates and enhances posture [35, 36]. However, these methods often involve high costs, limiting accessibility for the average consumer. Furthermore, manual therapy requires professional intervention, which adds to the burden on the healthcare system [37]. The SS technique utilized in this study requires only a single exercise rope to complete all actions. After brief instructions, individuals can independently perform the exercises at home, without the need for extensive space. These advantages sufficiently highlight the simplicity, environmental feasibility, and self-practice potential of the SS technique. Therefore, its simplicity and minimal environmental limitations make it an excellent choice for self-practice and improving body posture conditions.

More importantly, the SS technique provides a simple and effective solution from a holistic perspective to prevent issues related to abnormal body postures. By integrating and coordinating information across the body, a series of targeted exercises derived from the SS technique can be developed to prevent musculoskeletal conditions associated with poor posture. Clinicians may consider incorporating these exercises into routine rehabilitation or physical therapy practices. Educators can also integrate these simple movements into physical education classes, benefiting from their safety and effectiveness.

The application of the Exbody system was another advantage of this study. This system has been used in previous studies to assess changes in ten posture angles in postpartum women [23]. This system can reduce human measurement error and possesses certain accuracy and reliability. In this study, the Exbody system was used to simultaneously measure 11 indicators related to human posture, allowing researchers to no longer be limited to a single body part and to gain a systematic and comprehensive perspective on understanding human posture. In future research, the importance of the SS technique in human posture correction will be comprehensively discussed through the three aspects of etiology, signs, and function, combined with visual analogue scales, quality of life assessments, and the activity of human joints.

### Limitations and prospects

The study presented here has several limitations. The first limitation is that the intervention evaluation lasted only four days. Future studies should extend the observation time to assess the sustainability of the intervention technique. The second limitation is the lack of a control group. In future studies, a non-intervention control group and a positive control group (e.g., other common interventions like Pilates or McKenzie therapy) will be added to provide a more comprehensive understanding of the effects of the SS technique on body posture correction. Thirdly, the sample is predominantly female, and different age groups have not been considered, which may affect its generalizability. Additionally, the observation indicators are singular and lack subjective measures, such as pain scales and quality of life assessments. In future research, a wider range of indicators and studies will be applied to different genders and age groups to examine the impact of the intervention, which could provide valuable information for tailored clinical treatments.

## Conclusion

The SS technique, as a widely applicable and low-cost intervention, has the potential to improve body posture. It can become an effective fitness exercise, widely used in daily life, to prevent abnormal body posture from developing into low back pain or other musculoskeletal conditions.

## Data Availability

The datasets used and analyzed during the current study are available from the corresponding author upon reasonable request.

## Abbreviations

LBP: low back pain
SS technique: Spiral stabilization muscle chain technique
HAH: Horizontal alignment of head
HAA: Horizontal alignment of acromions
HAASIS: Horizontal alignment of anterior superior iliac spine
HAP: Horizontal alignment of patella
VAH: Vertical alignment of head
VAS: Vertical alignment of shoulder
PA: Pelvic angle
KA: Knee angle
FD: Front deviation
LD: Lateral deviation
OD: Overall deviation
